# Temporal trend of diarrhea morbidity rate with climate change: Egypt as a case study

**DOI:** 10.1007/s11356-022-22431-z

**Published:** 2022-08-17

**Authors:** Amal Saad-Hussein, Mona Adel Helmy, Lamia Samir Ellaithy, Ali Wheida, Mostafa El Nazer, Stephane C. Alfaro, Guillaume Siour, Agnes Borbon, Mohamed Magdy Abdel Wahab, Amira N. Mostafa

**Affiliations:** 1 Environmental & Occupational Medicine Department, Environment & Climate Change Research Institute, National Research Centre, El-Buhouth Street, Dokki, Cairo Egypt; 2 Theoretical Physics Department, Physical Research Institute, National Research Centre, El-Buhouth Street, Dokki, Cairo Egypt; 3 Laboratoire Inter-Universitaire Des Systèmes Atmosphériques, CNRS/Université de Paris-Est Créteil/Université de Paris-Diderot/IPSL, Créteil, France; 4 Laboratoire de Météorologie Physique, Université Clermont Auvergne, Aubière, France; 5 Astronomy and Meteorology Department, Faculty of Science, Cairo University, Giza, Egypt; 6 Egyptian Meteorological Authority, Abbasia, Cairo Egypt

**Keywords:** Climate Change, Diarrhea, Meteorological parameters, Extreme atmospheric temperature, Extreme precipitation

## Abstract

Many studies have detected a relationship between diarrhea morbidity rates with the changes in precipitation, temperature, floods, droughts, water shortage, etc. But, most of the authors were cautious in their studies, because of the lack of empirical climate-health data and there were large uncertainties in the future projections. The study aimed to refine the link between the morbidity rates of diarrhea in some Egyptian governorates representative of the three Egyptian geographical divisions with the meteorological changes that occurred in the 2006–2016 period for which the medical data are available, as a case study. Medical raw data was collected from the Information Centre Department of the Egyptian Ministry of Health and Population. The meteorological data of temperature and precipitation extremes were defined as data outside the 10th–90th percentile range of values of the period of study, and their analysis was done using a methodology similar to the one recommended by the WMO and integrated in the CLIMDEX software. Relationships between the morbidity rates of diarrhea in seven Egyptian governorates and the meteorological changes that occurred in the period 2006 to 2016 were analyzed using multiple linear regression analysis to identify the most effective meteorological factor that affects the trend of morbidity rate of diarrhea in each governorate. Statistical analysis revealed that some meteorological parameters can be used as predictors for morbidity rates of diarrhea in Cairo, Alexandria, and Gharbia, but not in Aswan, Behaira, and Dakahlia where the temporal evolution cannot be related with meteorology. In Red Sea, there was no temporal trend and no significant relationships between the diarrhea morbidity rate and meteorological parameters. The predictor meteorological parameters for morbidity rates of diarrhea were found to be depending on the geographic locations and infrastructures in these governorates. It was concluded that the meteorological data that can be used as predictors for the morbidity rate of diarrhea is depending on the geographical location and infrastructures of the target location. The socioeconomic levels as well as the infrastructures in the governorate must be considered confounders in future studies.

## Introduction

Climate change is an emerging public health emergency. The direct health impacts of climate change are associated with the increase in the frequency and intensity of heat waves, extreme precipitation events, floods, droughts, and fires. Attention has also been paid to indirect effects related to environmental disturbances such as crop failures, shifting patterns of disease vectors, and increases in the burden of diarrheal disease (Smith et al. 2014). Many national and international reports from the Egyptian Environmental Affairs Agency (EEAA), as well as international publications, proved that Egypt is vulnerable to climate change. Some predicted hazards related to climate change in Egypt include floods, droughts, and water shortage. The salinization of underground water and estuaries in coastal areas resulting from the rising of the sea level may cause contamination of public water supplies and encourage unhygienic practices (Egypt Third National Communication 2016). Moreover, considering both RCP4.5 and RCP8.5 scenarios, the daily maximal and minimal temperatures were increased by 1.3 ± 0.1 and 1.3 ± 0.3 °C, respectively, during the period 2006–2016, and about 80% of the days in a year would be hotter than the 90th percentile of the reference period (2006–2015), and the annual precipitation detected to have a significant decrease (Mostafa et al. 2019).

In a context of generalized climate warming, the Mediterranean and, particularly, its east basin are “hot spots” of climate change (Kim et al. 2019), which means that the surrounding countries will undergo in the next decade dramatic environmental changes to which they will have to adapt. In addition, the megacities of the area constitute hot spots of air pollution affecting air quality, climate, and ecosystems not only locally but also at the largest scale of the whole East Mediterranean region (Myriokefalitakis et al. 2011).

There is much evidence that the incidence of water- and foodborne illnesses can be affected by climate change, and this is linked to the fact that warm temperatures favor bacterial growth. Moreover, the WHO reported that diarrheal diseases are directly influenced by climate change due to the occurrence and the survival of bacterial agents, toxic algal blooms in water, and viral pathogens, in addition to lack of safe water that can compromise hygiene (WHO 2012).

Several studies have been carried out to identify how diarrhea rates change with the changes in precipitation, temperature, floods, droughts, water shortage, etc. Chou et al. (2010) found that changes in the maximum temperature and extreme rainfall were strongly related to diarrhea-associated morbidity, especially among children (0–14 years) and the elderly (40–64 years). A study in China revealed that diarrhea incidence increased with increase in temperature and relative humidity (Yang et al. 2021). The latter authors also found that a 1 °C rise in temperature increases the rates of diarrhea cases by 5.6%. A recent study in Nepal estimated an increased risk of 4.4% in diarrheal disease cases among children under 5 years of age per a 1 °C rise in mean temperature (Dhimal et al. 2022). Similar positive associations between temperature and diarrhea have been reported from Latin America and Africa (Musengimana et al. 2016; Thiam et al. 2017; Checkley 2000). Several previous studies considered weather variables only, although other factors such as water supply and sanitation, population density, socioeconomic status, and level of development may play an important role in predicting the occurrence of diarrhea (Gasana et al. 2022). Despite the published results, most of the authors were cautious in their studies. Because of the lack of empirical climate-health data, there were large uncertainties in the future projections.

This study aims to refine the link between the morbidity rates of diarrhea in some Egyptian governorates representative of the three Egyptian geographical divisions with the meteorological changes that occurred in the 2006–2016 period for which the medical data are available, as a case study. The specific objectives of the present study were to (1) quantify the annual trends of the diarrhea morbidity rate in Egypt for the period of the availability of the medical data (from the years 2006 to 2016) and (2) identify the most significant meteorological parameters affecting diarrhea in seven representative governorates.

## Materials and methods

### Study design

**Fig. 1:**
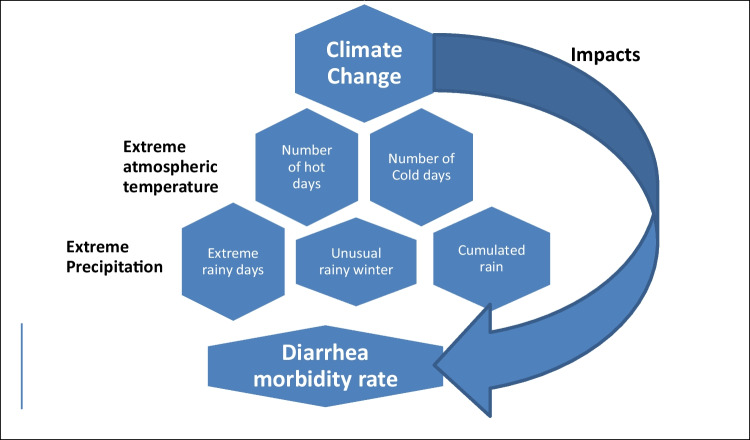
Study design

This study is a retrospective descriptive study of the diarrhea morbidity rate in some Egyptian governorates representative of the three Egyptian geographical divisions with the meteorological changes that occurred in the 2006–2016 period (Fig. 1).

### Setting of the study

Egypt has around 27 governorates, themselves divided into 3 divisions according to climate and social criteria: Upper Egypt, Lower Egypt, and Frontier. Aswan is representing Upper Egypt; Behaira, Dakahlia, and Gharbia represent Lower Egypt; and Alexandria and the Red Sea represent Frontier. Greater Cairo consists of three governorates (capital Cairo, Giza, and Qalubia). Moreover, Greater Cairo is considered one of the most densely populated megacities in the world.

### Meteorological data

In this study, we use the assimilated ERA-Interim (Berrisford et al. 2011) daily data (maximal and minimal temperatures, precipitations) of the period 2006–2016 for which the parallel medical data are available. The meteorological observations have been performed routinely for decades by the EMA in its network of surface stations. As these data were not distributed to the large public and therefore rarely used, an effort was made recently to check the quality of the hourly meteorological measurements performed from 1 January 2004 to 31 December 2010 at 8 of the EMA stations (Korany et al. 2016). These high-quality data were then deposited in a public repository (https://www.pangaea.de/) from which they can be downloaded at no cost. The datasets contain the hourly values of the air temperature from which the maximum temperature (*Tmax*) and minimum temperature (*Tmin*) can be extracted, but not the precipitation. In this study, we have not retained the stations located in areas with a very low population density and only selected governorates. Note that this consistency is not ensured for variables that are not assimilated but modeled, such as temperature and precipitation extremes (Sillmann et al. 2013).

Therefore, reanalysis uses a forecast model to assimilate and compare observations of various types and from multiple sources, thus becoming able to extrapolate information from locally observed parameters to unobserved parameters at nearby locations (Dee et al. 2011). The daily precipitation and *Tmax* and *Tmin* at 2 m necessary for this study were extracted using the ECMWF web user interface (http://apps.ecmwf.int/datasets/data/interim-full-daily/levtype¼sfc/) for the included governorates from 2006 to 2016. As shown in a companion paper (Mostafa et al. 2019), sub-regional differences of climate variability can be evidenced in Egypt. Temperature and precipitation extremes are defined as data outside the 10th–90th percentile range of values of the period of study (Frich et al. 2002), and their analysis is done using a methodology similar to the one recommended by the WMO and integrated in the CLIMDEX software (Donat et al. 2013).

### Medical data

The raw medical data for the 2006–2016 periods were collected from the Information Centre Department of the Egyptian Ministry of Health and Population (MOHP). The yearly population data for each governorate were obtained from the data published in the CAPMUS reports. The yearly diarrhea morbidity rates of each governorate were obtained by dividing the number of cases of diarrhea recorded by MOHP by the total population, and multiplying these results by 100,000 yields the yearly prevalence of diarrhea in 100,000 populations in each governorate. These calculated values of the diarrhea prevalence were verified by comparison with the data published for the whole Egypt in the WHO annual statistical reports.

### Statistical analysis

**Fig. 2:**
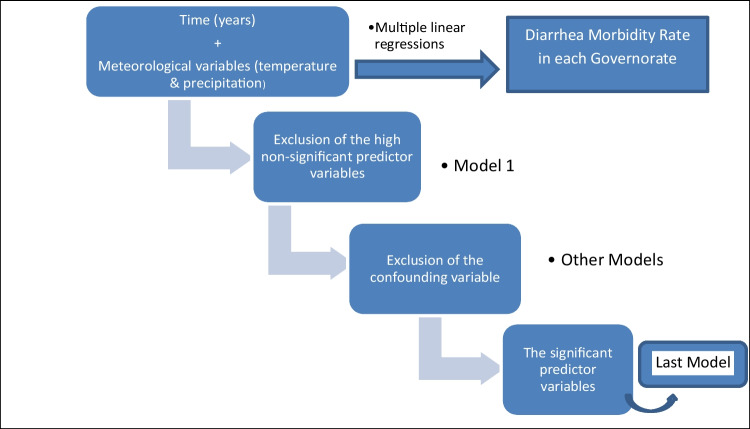
Statistical analysis

The collected data and the calculated morbidity rates were digitalized. Statistical analysis was done through SPSS version 20. Pearson’s correlation coefficient was used to quantify the temporal trend of the incidence of diarrhea and the relationships between this incidence and the meteorological parameters. The multiple linear regressions were used to identify the most effective meteorological parameters, in the form of the effects of time in years and the meteorological variables (extreme hot and cold days, unusual rainy winters, extreme rainy days, and cumulative rain), that affect the trend of the morbidity rate of diarrhea in each governorate (Fig. 2).

## Results

**Fig. 3:**
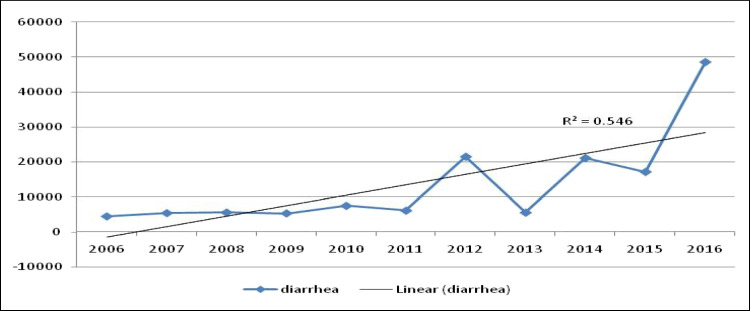
Trend of diarrhea morbidity rate in Egypt during the period 2006–2016

Figure 3 shows the trend of the diarrhea morbidity rate in Egypt between 2006 and 2016, which was significantly increasing with the time during the period 2006–2016 in Egypt (*r* = 0.4, *P* < 0.05). It increased slowly from 2006 to 2011, reached a high point in 2012, and then markedly increased in the year 2016.

**Fig. 4:**
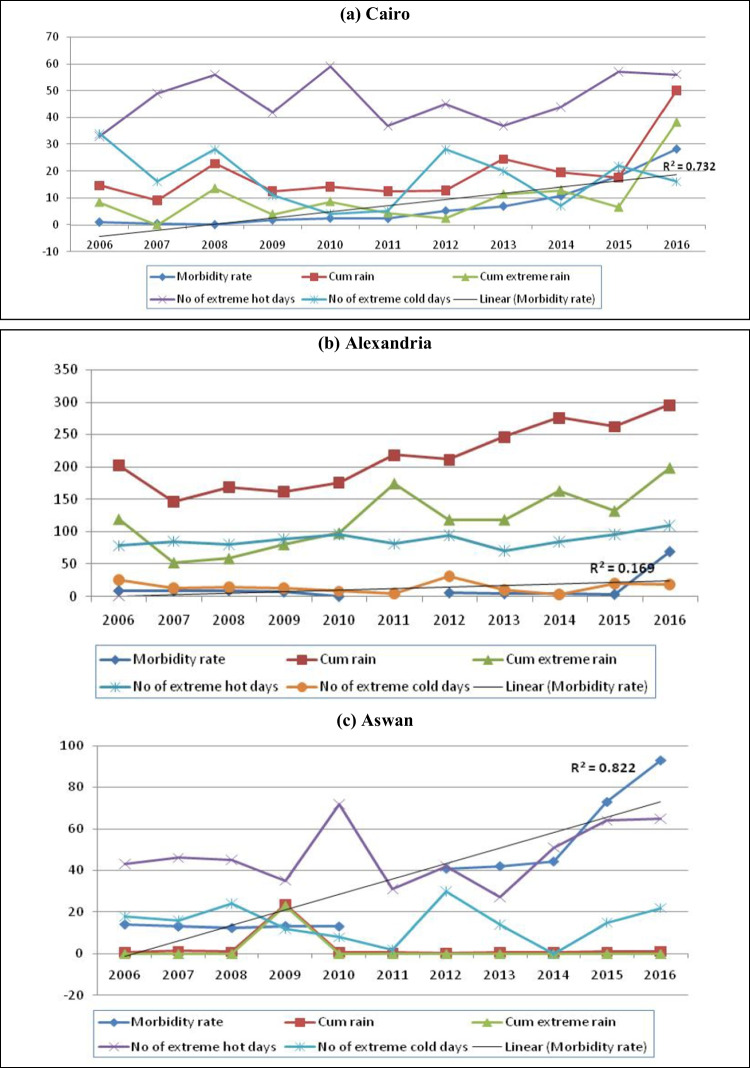

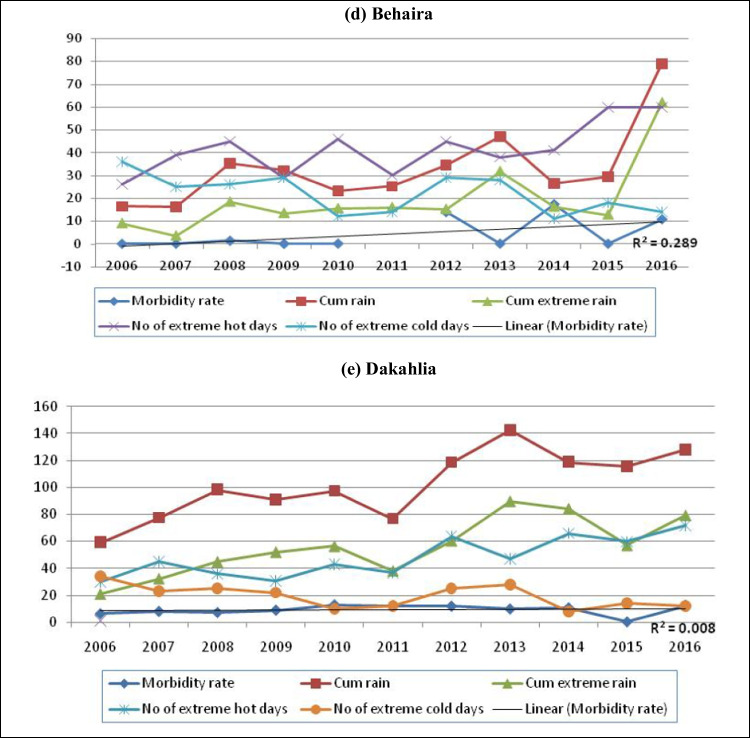

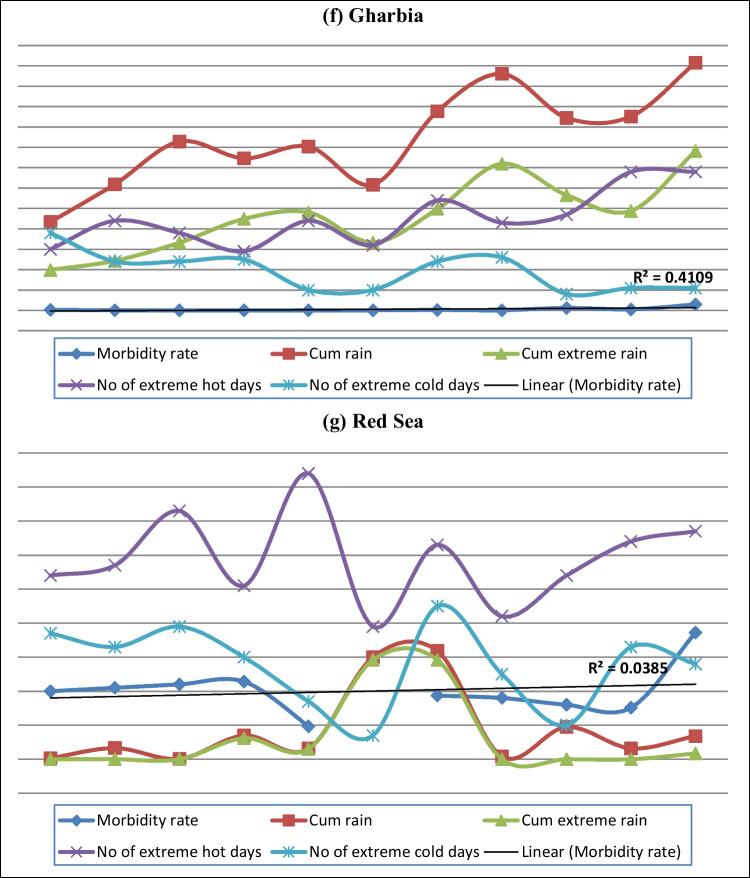
Trends of diarrhea morbidity rate and some meteorological factors in a selection of Egyptian governorates. **a** Cairo. **b** Alexandria. **c** Aswan. **d** Behaira. **e** Dakahlia. **f** Gharbia. **g** Red Sea

Figure 4 shows that the diarrhea morbidity rates were increased in most governorates, and suggests that, except in Aswan and the Red Sea, this increase was positively correlated with the cumulative rain, number of extreme rainy days, and number of extreme hot days.

The results of the statistical analysis (Table 1) show that there was a significant temporal increase of the number of extreme rainy days in Greater Cairo and of the number of hot days in Alexandria, Dakahlia, Behaira, and Gharbia, and a decrease of the number of cold days in Greater Cairo, Dakahlia, Behaira, and Gharbia. Conversely, there was no significant correlation between time and meteorological variables in Aswan and Red Sea Governorates.

**Table 1:**
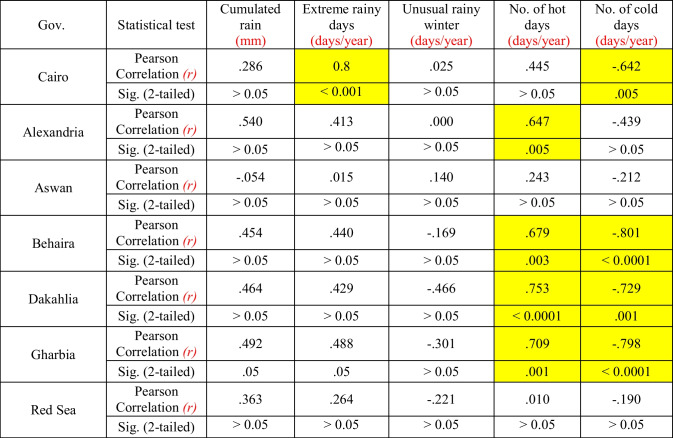
Relationship of the temporal trends of some meteorological variables in a selection of Egyptian governorates (the period considered is 2006–2016)

Table 2 shows that the incidence of diarrhea in Greater Cairo Governorate was significantly correlated with years, extreme rainy days, and unusual rainy winters in the statistical model (2 and 3). After the removal of the effects of the numbers of extreme cold and hot days (model 4), it becomes clear that the morbidity rate of diarrhea was significantly affected by the extreme rainy days and the number of unusually rainy winters.

**Table 2:**
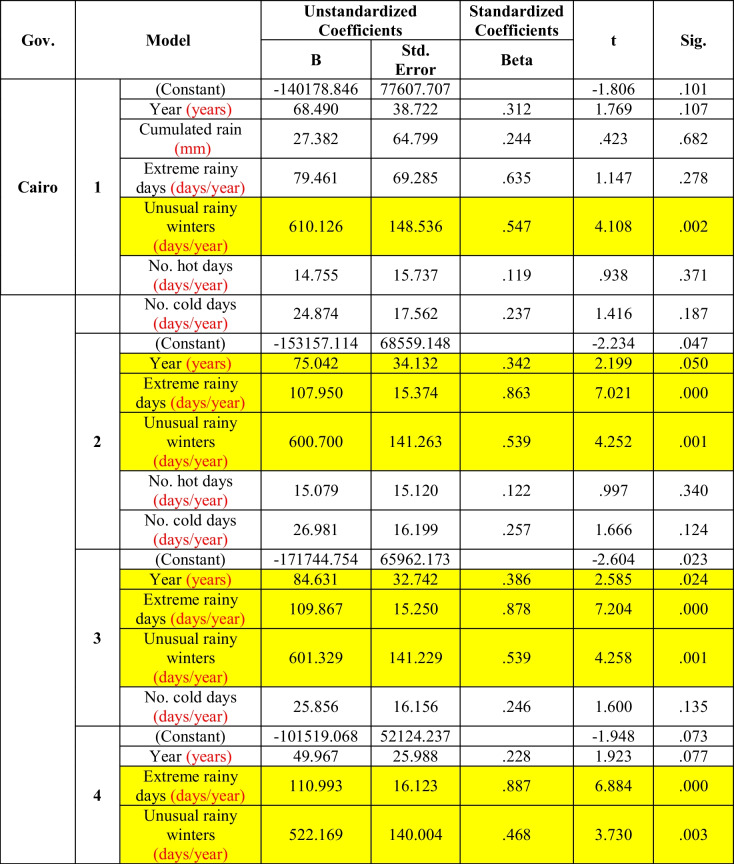
Multiple regression between diarrhea and climate factors in Greater Cairo (the period considered is 2006–2016)

In Alexandria (Table 3), the morbidity rate of diarrhea was significantly affected by the number of extreme hot days, and this significance increases after exclusion of the effects of the other meteorological factors and of time (model 6).

**Table 3:**
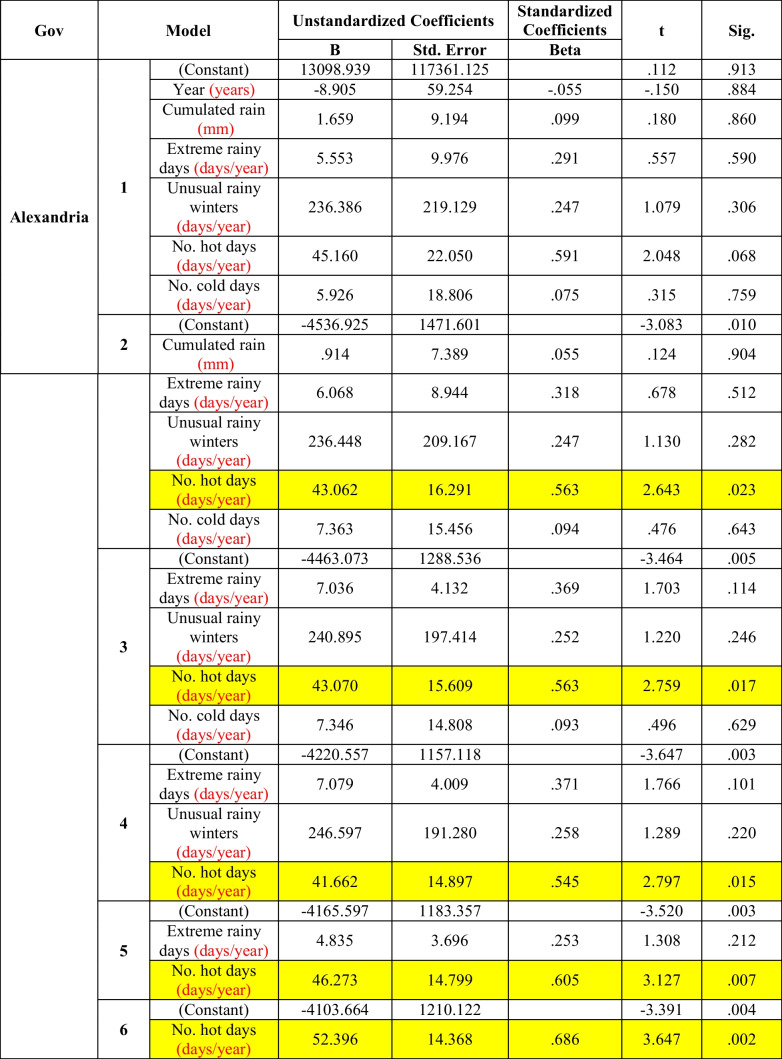
Multiple regression between diarrhea and climate factors in Alexandria (the period considered is 2006–2016)

In Aswan (Table 4), the morbidity rate of diarrhea was significantly affected by years only in all the models (models 1–5), and this significance increases with the removal of the meteorological factors. Therefore, there was no significant effect of the meteorological parameters on the incidence of diarrhea at this location.

**Table 4:**
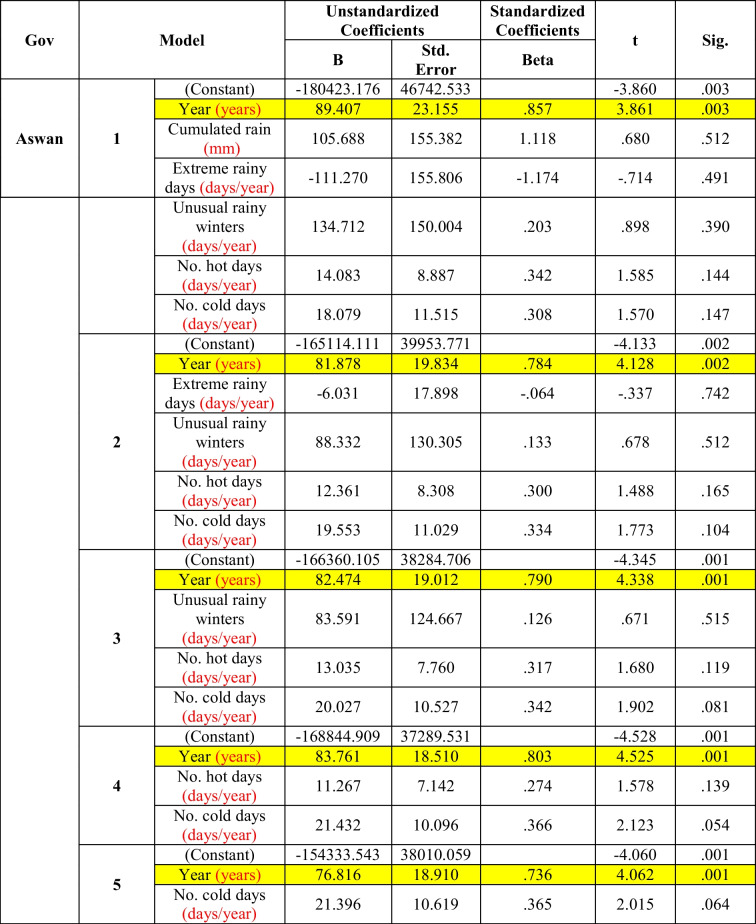
Multiple regression between diarrhea and climate factors in Aswan (the period considered is 2006–2016)

In Behaira also (Table 5), the morbidity rate of diarrhea was significantly affected by years only (models 5 and 6), and this effect became significant only after removal of the effects of the meteorological parameters. Thus, there was no significant effect of the meteorological parameters on the incidence of diarrhea.

**Table 5:**
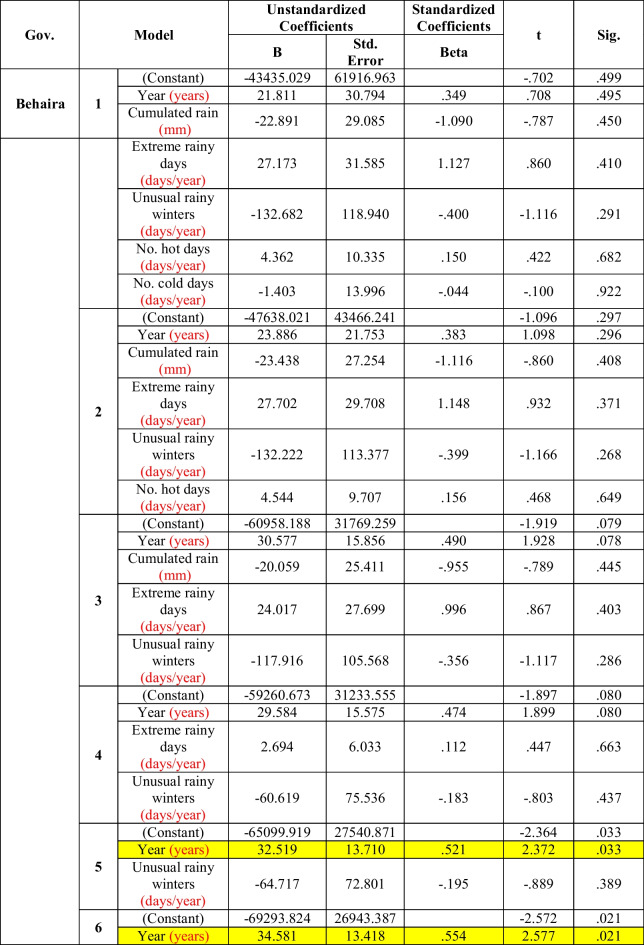
Multiple regression between diarrhea and climate factors in Behaira (the period considered is 2006–2016)

Table 6 shows that the morbidity rate of diarrhea was significantly affected by years only in all models (models 1–5) in Dakahlia Governorate, and this significant effect was increased with the removal of the effects of the meteorological parameters. Here also, there were no significant effects of the meteorological parameters on the incidence of diarrhea.

**Table 6:**
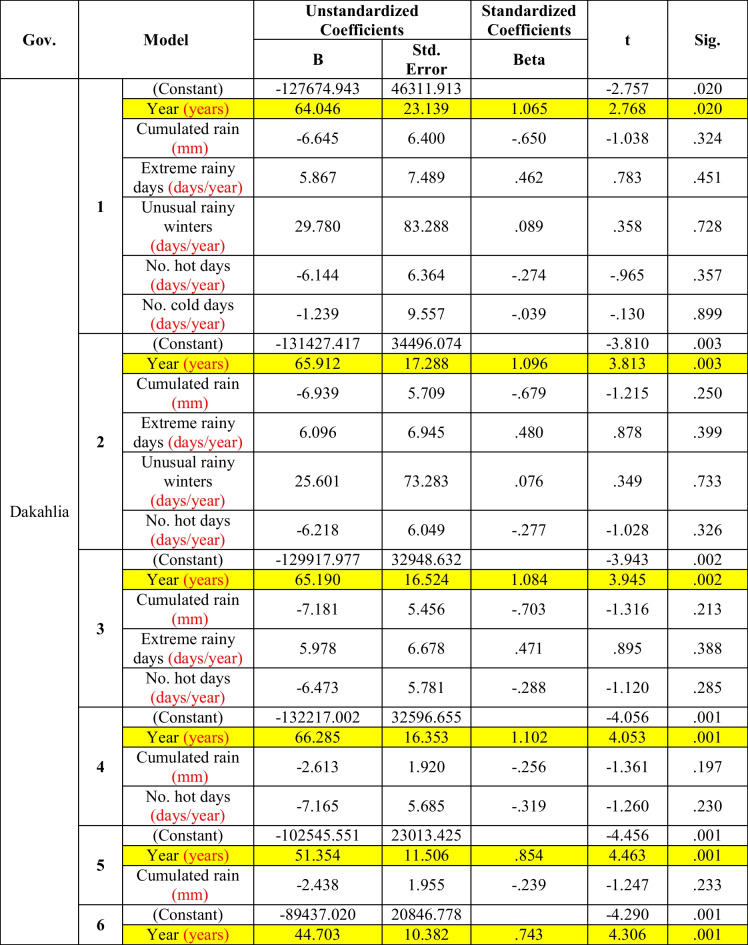
Multiple regression between diarrhea and climate factors in Dakahlia (the period considered is 2006–2016)

In Gharbia Governorate, the morbidity rate of diarrhea was significantly positively correlated with the number of extreme hot days (model 6), after exclusion of the statistical effects of the years and other meteorological factors (Table 7).

**Table 7:**
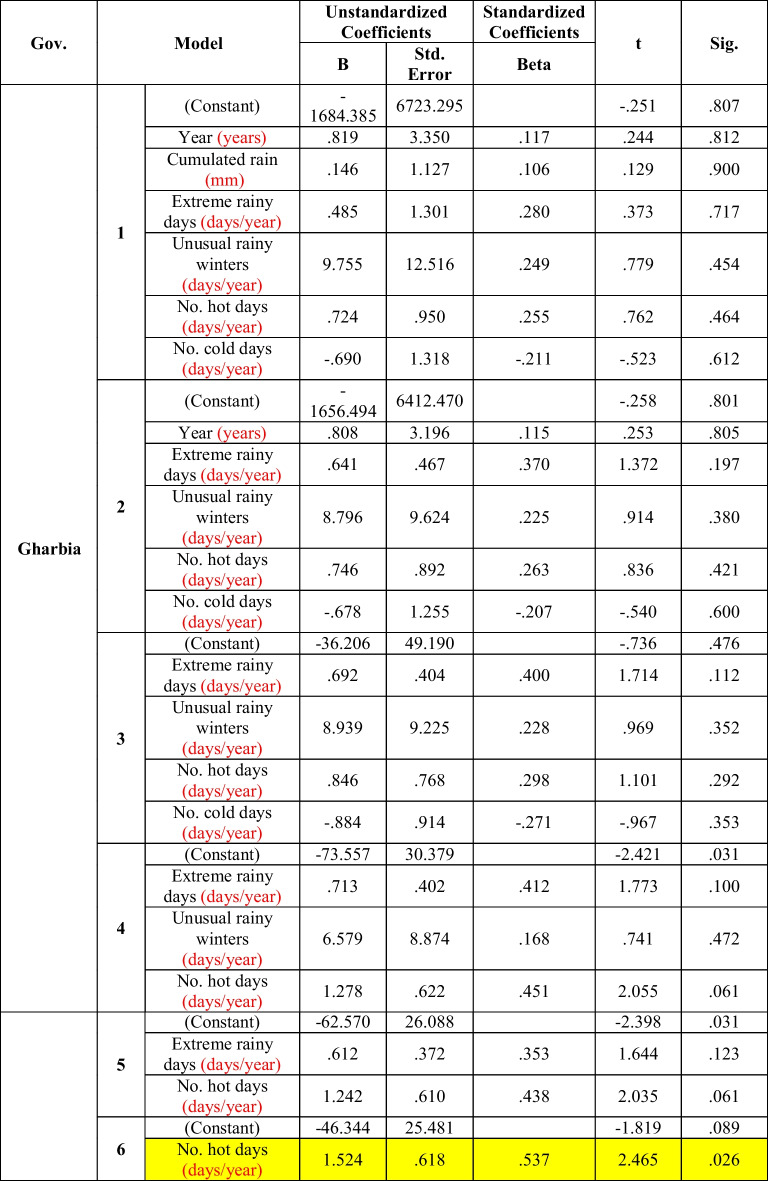
Multiple regression between diarrhea and climate factors in Gharbia (the period considered is 2006–2016)

Finally, in Red Sea Governorate, there was no significant correlation between the morbidity rate of diarrhea and either the years or the meteorological parameters (Table 8).

**Table 8:** Multiple regression between diarrhea and climate factors in Red Sea (the period considered is 2006–2016)

Gov	Model		Unstandardized coefficients		Standardized coefficients	*t*	Sig
			***B***	**Std. error**	**Beta**		
**Red Sea**	**1**	(Constant)	− 14,016.617	9577.823		− 1.463	.174
		Year (years)	6.990	4.785	.446	1.461	.175
		Cumulated rain (mm)	2.349	12.086	.286	.194	.850
		Extreme rainy days (days/year)	− 3.620	11.616	− .436	− .312	.762
		Unusual rainy winters (days/year)	− 19.854	30.232	− .238	− .657	.526
		No. hot days (days/year)	.040	2.169	.006	.019	.985
		No. cold days (days/year)	.857	2.346	.109	.365	.722
	**2**	(Constant)	− 14,040.004	9053.634		− 1.551	.149
		Year (years)	7.003	4.515	.447	1.551	.149
		Cumulated rain (mm)	2.297	11.220	.280	.205	.841
		Extreme rainy days (days/year)	− 3.581	10.894	− .432	− .329	.749
		Unusual rainy winters (days/year)	− 20.060	26.824	− .241	− .748	.470
		No. cold days (days/year)	.868	2.158	.111	.402	.695
	**3**	(Constant)	− 14,736.461	8048.523		− 1.831	.092
		Year (years)	7.355	4.005	.469	1.836	.091
		Extreme rainy days (days/year)	− 1.395	2.068	− .168	− .674	.513
		Unusual rainy winters (days/year)	− 22.987	21.773	− .276	− 1.056	.312
		No. cold days (days/year)	.796	2.041	.101	.390	.704
	**4**	(Constant)	− 14,432.029	7744.824		− 1.863	.085
		Year (years)	7.213	3.856	.460	1.871	.084
		Extreme rainy days (days/year)	− 1.472	1.991	− .177	− .739	.473
		Unusual rainy winters (days/year)	− 20.049	19.749	− .241	− 1.015	.329
	**5**	(Constant)	− 12,898.078	7339.897		− 1.757	.101
		Year	6.447	3.654	.411	1.765	.099
		Unusual rainy winters (days/year)	− 20.809	19.400	− .250	− 1.073	.302
	**6**	(Constant)	− 14,656.196	7190.335		− 2.038	.060
		Year (years)	7.314	3.581	.466	2.042	.059

Table 9 summarizes the relationships of the diarrhea morbidity rate and the meteorological parameters. Extreme rainy days and the unusual rainy winter were significant predictors for the diarrhea morbidity rate in Cairo, while the number of hot days was a significant predictor for the morbidity rate in Alexandria and Gharbia Governorates. There were no meteorological predictors for diarrhea morbidity in the other governorates, while the time factor was a significant predictor for the morbidity rate of diarrhea in Aswan, Behaira, and Dakahlia.

**Table 9:**
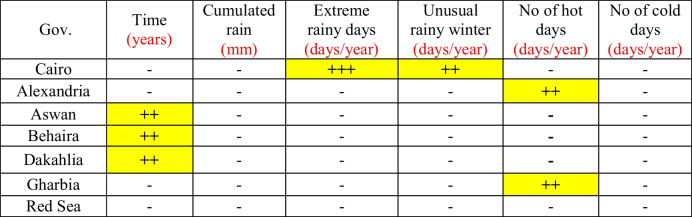
Summary of the results of multiple regression between diarrhea and climate factors the different governorates (the period considered is 2006–2016)

## Discussion

Climatic change and the increased number of associated extreme weather events have been shown to affect significantly seasonal diarrhea in susceptible populations. For instance, Hashizume et al. (2007) studied the association between climate variability and hospital visits for non-cholera diarrhea in Bangladesh and they found evidence that there was a relation between the frequency of the visits and climatic variations. Moreover, they expected that future climate change would exacerbate diarrhea. Diarrhea is a climate-sensitive health problem, and therefore expected to become worse in a changing climate (WHO Fact Sheet No. 266,2012; Egypt Third National Communication 2016).

The statistical analysis of the Egyptian medical data shows that the morbidity rate of diarrhea among children under 5 years decreased between 2000 and 2008 as a result of the application of the rehydration health campaign program (El-Zanaty and Way 2009; Mansour et al. 2013). However, there have been suggestions that climate change in Egypt would make this reduction in diarrhea morbidity rate difficult to maintain (Egypt Third National Communication 2016), and this particularly after the stop of the rehydration health campaign program. This may already explain the increase in the morbidity rate of diarrhea detected in the present study, and there is an expectation that this increase will continue as climate change proceeds.

There is much evidence that climate change has already affected Egypt, but its impacts differ from one Egyptian governorate to the other (Mostafa et al. 2019). These spatial differences are confirmed by the present study. In Greater Cairo, a significant increase of the number of extreme rainy days and a decrease of the number of cold days have been detected. The number of hot days was also found to increase significantly in Alexandria, Dakahlia, Behaira, and Gharbia, and the number of cold days to decrease in Dakahlia, Behaira, and Gharbia. However, there was no significant temporal trend of the meteorological variables in Aswan and Red Sea Governorates.

The hazards related to climate change in Egypt include floods, droughts, water shortage, and an increased salinity of groundwater and estuaries in coastal areas due to the sea level rise (SLR). This may cause contamination of public water supplies and encourage unhygienic practices (Egypt Third National Communication 2016). From Fig. 4, it can be seen that diarrhea morbidity rates have increased with the increase in cumulative rain, number of extreme rainy days, and number of extreme hot days in most governorates, except in Aswan and the Red Sea. This could be attributed to a climate effect because meteorological parameters did not change significantly in these two governorates.

In order to achieve the objective of our current study, backward linear regression models were applied to disentangle the effects of the climate variables on the diarrhea morbidity rate. The proposed models include a variety of climate factors (numbers of extreme hot, cold, and rainy days; unusual rainy winters; and cumulated rainfall per year) that have a potential influence on the time trends of diarrhea morbidity rates in our selection of Egyptian governorates.

The results of Chou et al. (2010) indicate that maximum temperature and extreme rainfall days have the strongest effect on diarrhea-associated morbidity. In the present study, the effects of meteorological factors on the diarrhea morbidity rate were found to be varied with the geographic location of the governorate. *In Greater Cairo*, the morbidity rate was significantly affected by the cumulative extreme rain and number of unusually rainy winter days after the removal of the effect of numbers of extreme cold and hot days (Table 2). Extreme events of unusual rainy winters may result in accumulation of water especially if there is no well-prepared infrastructure to manage it. These cumulative extra rains may lead to contamination of the sources of drinking water. Wolf et al. (2014) reported that extreme events of floods may flush contaminations into drinking water, deteriorating the quality of local water sources and increasing diarrheal risk. Moreover, Liu et al. (2018) defined that economic levels were modifiers for the impact of floods on diarrhea in the regions with low economic levels.

But, in *Alexandria* (Table 3) and *Gharbia* (Table 7), morbidity rates of diarrhea were significantly affected by the number of extreme hot days, and the significance of the correlation was increased after exclusion of the effects of the other meteorological factors as well as the time trend (model 6). This was explained by WHO Fact Sheet No. 266 (2012). It was reported as a fact that diarrheal diseases due to water- and foodborne diseases are directly affected by the rise in temperature due to climate change, which will affect the occurrence and the survival of bacterial agents, toxic algal blooms in water, and viral pathogens, and the lack of safe water for domestic uses and hygiene.

Chou et al. (2010) noted that the impact of the maximum temperature on diarrhea-associated morbidity was prominent among children (0–14 years) and older adults (40–64 years). These age groups are generally considered being the most vulnerable to climate change (Egypt Third National Communication 2016), but further investigation is still needed to determine exactly which age classes are the most at risk in Egypt.

In *Aswan* (Table 4), the diarrhea morbidity rate increased significantly with time only in all the models, from model 1 to model 5, and the significance was increased with the removal of the meteorological factors. Therefore, the meteorological parameters in this governorate proved to have no particular role on the morbidity rate of diarrhea, but this could be due simply to the fact that no significant change of these parameters was detected during the studied period.

In *Behaira*, the time trend was the only significant predictor to the morbidity rate of diarrhea in model 5 and model 6, and this significance was not detected in models 1 to 4 after the removal of the effects of the meteorological variables (Table 5). Therefore, climatic variables were considered confounders decreasing the significant predictor effect of the time trend for the morbidity rate of diarrhea in *Behaira*. Therefore, the significant increase of extreme hot days and the significant decrease in the extreme cold days are not the main predictor for diarrheal morbidity rates in this Egyptian governorate. Other confounders, such as the availability of health facilities in the form of stopping of rehydration program, sanitation, hygiene, and illiteracy, may have a significant role in the significant trend of diarrhea (*R*^2^ = 0.289) (Fig. 4d). These factors could be of great effect in this governorate, but this study was unable to include this data due to the lack of proper reporting and recording of such variables, which can be collected by surveys in further studies.

Although the diarrhea morbidity rate seemed to be related to cumulative rain, extreme rainy days and extreme hot days in Dakahlia (Fig. 4e), multiple linear regression revealed that the time trend was the only significant predictor for the diarrhea morbidity rate in all the models (Table 6). From model 1 to model 3, the significance of the correlation increased with the removal of the statistical effect of the meteorological parameters: cumulative extreme rain, unusual rainy winters, and extreme cold days. Cumulative rain and extreme hot days in model 4 and cumulative rain in model 6 did not change the level of significant level of time trend on the diarrhea. The same results were detected by Bhandari et al. (2012), who found that atmospheric temperature was not a predictor of diarrheal occurrence in time series analysis, although they observed that higher cases of diarrhea were recorded during the hot periods. They attributed the misleading results to the presence of other confounders not related to climatic factors, such as sanitation, hygiene, availability of health facilities, and illiteracy.

These factors could also play a role in the present study. They will definitely need to be investigated further on.

In *Red Sea* Governorate, neither the time trend nor the meteorological parameters proved to be significant predictors for diarrhea morbidity rate (Table 8). It is clear that apart from a severe increase in diarrhea cases in 2015/2016 (Fig. 4g), there were no variations in the morbidity rate of diarrhea in Red Sea Governorate between 2006 and 2016. Moreover, in the same period, there was no significant temporal trend of the meteorological parameters but only huge variations (Table 1). This could explain the absence of any predictor for the diarrhea morbidity rate in this governorate.

Therefore, the geographic location of the different governorates was identified as an effective parameter for the impact of climate change on the morbidity rates of diarrhea in Egypt. Moreover, the impact of climate change in governorates with low socioeconomic levels could not be evidenced, probably because of such confounders as the lesser availability of health facilities, safe water supply, and sanitation.

## Conclusion

Therefore, it was concluded that the meteorological data that can be used as predictors for the evolution of diarrhea morbidity rate differ with the geographic location of the governorate in Egypt. Moreover, the socioeconomic levels as well as the infrastructures in the governorate will need to be considered confounders in the following studies.

Since climatic changes have contributed to the occurrence of diarrhea in some Egyptian governorates, it is necessary to develop an early warning system based on climate change information to manage disease control. A retrospective longitudinal study is recommended including primary data for drinking water sources, sanitation, sociocultural factors, availability of health services, and diarrhea control programs, as well as climatic variables.

## Data Availability

The datasets analyzed during the current study are available from the corresponding author on reasonable request.
