# Correction to: Temporal trend of diarrhea morbidity rate with climate change: Egypt as a case study

**DOI:** 10.1007/s11356-023-25225-z

**Published:** 2023-01-09

**Authors:** Amal Saad‑Hussein, Mona Adel Helmy, Lamia Samir Ellaithy, Ali Wheida, Mostafa El Nazer, Stephane C. Alfaro, Guillaume Siour, Agnes Borbon, Mohamed Magdy Abdel Wahab, Amira N. Mostafa

**Affiliations:** 1 Environmental & Occupational Medicine Department, Environment & Climate Change Research Institute, National Research Centre, El‑Buhouth Street, Dokki, Cairo, Egypt; 2 Theoretical Physics Department, Physical Research Institute, National Research Centre, El‑Buhouth Street, Dokki, Cairo, Egypt; 3 Laboratoire Inter‑Universitaire Des Systèmes Atmosphériques, CNRSUniversité de Paris-Est CréteilUniversité de Paris-Diderot/IPSL, Créteil, France; 4 Laboratoire de Météorologie Physique, Université Clermont Auvergne, Aubière, France; 5 Astronomy and Meteorology Department, Faculty of Science, Cairo University, Giza, Egypt; 6 Egyptian Meteorological Authority, Abbasia, Cairo, Egypt

**Correction to: Environmental Science and Pollution Research**

**https://doi.org/10.1007/s11356-022-22431-z**

The correct family name of the 5^th^ is El Nazer.

The Original article has been corrected.

